# (*Z*)-2-(5-Chloro-2-oxoindolin-3-yl­idene)-*N*-phenyl­hydrazinecarbothio­amide

**DOI:** 10.1107/S1600536811044199

**Published:** 2011-11-02

**Authors:** Amna Qasem Ali, Naser Eltaher Eltayeb, Siang Guan Teoh, Abdussalam Salhin, Hoong-Kun Fun

**Affiliations:** a School of Chemical Sciences, Universiti Sains Malaysia, Minden, Penang, Malaysia; b Faculty of Science, Sabha University, Libya; c Department of Chemistry, International University of Africa, Khartoum, Sudan; d X-ray Crystallography Unit, School of Physics, Universiti Sains Malaysia, 11800 USM, Penang, Malaysia

## Abstract

In the title compound, C_15_H_11_ClN_4_OS, the dihedral angle between the nine-membered 5-chloro­indolin-2-one ring system and the benzene ring is 10.00 (6)°. Intra­molecular cyclic N—H⋯O and C—H⋯S hydrogen-bonding inter­actions [graph set *S*(6)] are present in the N—N—C—N chain between the ring systems. In the crystal, mol­ecules form centrosymmetric cyclic dimers through inter­molecular N—H⋯O hydrogen bonds [graph-set *R*_2_^2^(8)] and are extended by C—H⋯Cl inter­actions into infinite chains which propagate along [100].

## Related literature

For related structures, see: Ferrari *et al.* (2002 ▶); Pervez *et al.* (2010 ▶); Ramzan *et al.* (2010 ▶). For various biological activities of Schiff bases, see: Bhandari *et al.* (2008 ▶); Bhardwaj *et al.* (2010 ▶); Pandeya *et al.* (1999 ▶); Sridhar *et al.* (2002 ▶); Suryavanshi & Pai (2006 ▶). For cytotoxic and anti­cancer activities of isatin and its derivatives, see: Vine *et al.* (2009 ▶). For bond-length data, see; Allen *et al.* (1987 ▶). For graph-set analysis, see Bernstein *et al.* (1995 ▶).
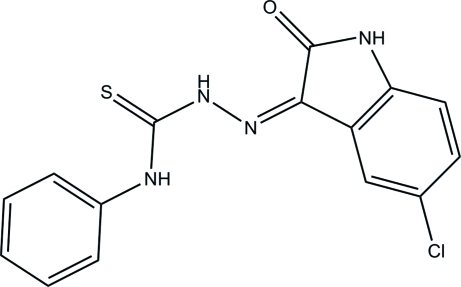


## Experimental

### 

#### Crystal data

C_15_H_11_ClN_4_OS
*M* *_r_* = 330.79
Monoclinic, 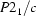

*a* = 5.7117 (2) Å
*b* = 17.9510 (7) Å
*c* = 14.2455 (5) Å
β = 91.262 (2)°
*V* = 1460.25 (9) Å^3^
*Z* = 4
Mo *K*α radiation
μ = 0.41 mm^−1^
*T* = 100 K
0.53 × 0.16 × 0.13 mm

#### Data collection

Bruker APEXII CCD diffractometer
Absorption correction: multi-scan (*SADABS*; Bruker, 2005 ▶) *T*_min_ = 0.812, *T*_max_ = 0.950
15819 measured reflections
4183 independent reflections
3424 reflections with *I* > 2σ(*I*)
*R*_int_ = 0.044

#### Refinement

*R*[*F* ^2^ > 2σ(*F* ^2^)] = 0.040
*wR*(*F* ^2^) = 0.101
*S* = 1.04
4183 reflections
211 parameters
H atoms treated by a mixture of independent and constrained refinement
Δρ_max_ = 0.51 e Å^−3^
Δρ_min_ = −0.23 e Å^−3^

### 

Data collection: *APEX2* (Bruker, 2005 ▶); cell refinement: *SAINT* (Bruker, 2005 ▶); data reduction: *SAINT*; program(s) used to solve structure: *SHELXS97* (Sheldrick, 2008 ▶); program(s) used to refine structure: *SHELXL97* (Sheldrick, 2008 ▶); molecular graphics: *SHELXTL* (Sheldrick, 2008 ▶); software used to prepare material for publication: *SHELXTL* and *PLATON* (Spek, 2009 ▶).

## Supplementary Material

Crystal structure: contains datablock(s) I, global. DOI: 10.1107/S1600536811044199/zs2150sup1.cif

Structure factors: contains datablock(s) I. DOI: 10.1107/S1600536811044199/zs2150Isup2.hkl

Supplementary material file. DOI: 10.1107/S1600536811044199/zs2150Isup3.cml

Additional supplementary materials:  crystallographic information; 3D view; checkCIF report

## Figures and Tables

**Table 1:** Hydrogen-bond geometry (Å, °)

*D*—H⋯*A*	*D*—H	H⋯*A*	*D*⋯*A*	*D*—H⋯*A*
N1—H1*N*1⋯O1^i^	0.89 (2)	1.98 (2)	2.8560 (17)	171 (2)
N3—H1*N*3⋯O1	0.86 (2)	2.08 (2)	2.7563 (16)	135.9 (19)
C2—H2*A*⋯Cl1^ii^	0.93	2.81	3.6935 (17)	158
C11—H11*A*⋯S1	0.93	2.61	3.2423 (14)	126

Symmetry codes: (i) 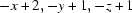; (ii) 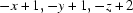.

**Fig. 1.**
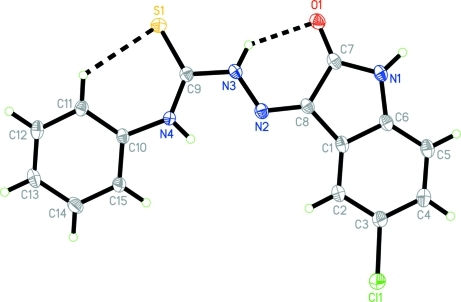
The molecular structure of the title compound, with 50% probability displacement ellipsoids and the atom-numbering scheme.

**Fig. 2.**
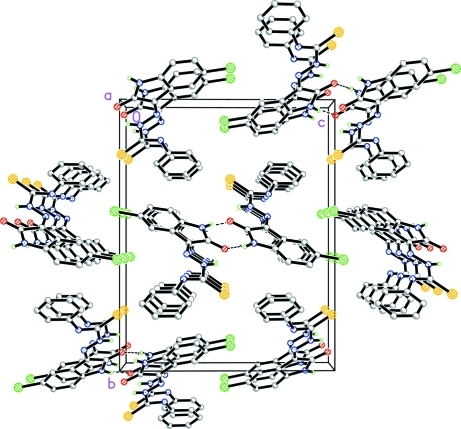
The crystal packing of the title compound viewed down the a axis. Hydrogen bonds are shown as dashed lines.

## supplementary crystallographic information

### Comment

Isatin (2,3-dioxindole) is an endogenous compound identified in humans, and its effect has been studied in a variety of systems (Vine *et al.*, 2009). Biological properties of isatin and its derivatives include a range of actions in the brain and offer protection against certain types of infections, such as anti-bacterial (Suryavanshi & Pai, 2006) antifungal, anticonvulsant, anti-HIV (Pandeya *et al.*,1999), anti-depressant and anti-inflammatory activities (Bhandari *et al.*, 2008). In this paper we describe the single-crystal X-ray diffraction study of the title compound, C_15_H_11_ClN_4_OS.

In this compound (Fig. 1), the dihedral angle between the nine-membered 5-chloroindolin-2-one ring system and benzene ring is 10.00 (6)°. Atoms C8 in the 5-chloroindolin-2-one ring and C10 in the benzene ring are joined by a chain of four atoms (N2/N3/C9/N4) giving a torsion angle 7.47 (19)° while the the torsion angles C8—N2—N3—C9 and C10—N4—C9—N3 are 173.15 (13)° and -178.02 (13)°, respectively. The essentially planar conformation of the molecule is maintained by cyclic intramolecular N3—H···O1 and C11—H···S1 hydrogen-bonding interactions [graph set *S*(6) (Bernstein *et al.*, 1995)] (Table 1) together with an *S*(5) N3—H···N2 interaction.

In the crystal the molecules form centrosymmetric cyclic dimers through intermolecular N—H···O hydrogen bonds [graph set *R*^2^_2_(8)] and are extended by C—H···Cl interactions into infinite chains which propagate along [100] (Fig. 2). Weak C—H···π interactions are also present: C5—H5A···*Cg3*^iii^ = 3.6321 (18) Å, where *Cg3*^iii^ is the centroid of the C10—C15 ring [symmetry code: (iii) -*x* + 1, *y* + 1/2, -*z* + 3/2].

### Experimental

The Schiff base have been synthesized by refluxing the reaction mixture of hot ethanolic solution (30 ml) of 4-phenyl-3-thiosemicarbazide (0.01 mol) and hot ethanolic solution (30 ml) of 5-chloroisatin (0.01 mol) for 2 h. The precipitate formed during reflux was filtered, washed with cold ethanol and recrystallized from hot ethanol: yield 97%; m.p. 521.4-521.9 K). The orange crystals were grown in acetone-DMF (3:1) by slow evaporation at room temperature.

### Refinement

N bound H atoms were located in a difference Fourier map and were refined freely. The remaining H atoms were positioned geometrically and refined using a riding model with C–H = 0.93 Å and *U*_iso_(H) = 1.2*U*_eq_(C). The highest residual electron density peak (0.510 eÅ^-3^ is located at 0.87 Å from C3 and the deepest hole (-0.228 eÅ^-3^) is located at 0.56 Å from Sl.

### Crystal data

**Table:**

C_15_H_11_ClN_4_OS	*F*(000) = 680
*M**_r_* = 330.79	*D*_x_ = 1.505 Mg m^−^^3^
Monoclinic, *P*2_1_/*c*	Melting point = 521.3–521.9 K
Hall symbol: -P 2ybc	Mo *K*α radiation, λ = 0.71073 Å
*a* = 5.7117 (2) Å	Cell parameters from 6388 reflections
*b* = 17.9510 (7) Å	θ = 2.3–29.8°
*c* = 14.2455 (5) Å	µ = 0.41 mm^−^^1^
β = 91.262 (2)°	*T* = 100 K
*V* = 1460.25 (9) Å^3^	Block, orange
*Z* = 4	0.53 × 0.16 × 0.13 mm

### Data collection

**Table:**

Bruker APEXII CCD diffractometer	4183 independent reflections
Radiation source: fine-focus sealed tube	3424 reflections with *I* > 2σ(*I*)
graphite	*R*_int_ = 0.044
φ and ω scans	θ_max_ = 29.9°, θ_min_ = 1.8°
Absorption correction: multi-scan (*SADABS*; Bruker, 2005)	*h* = −7→7
*T*_min_ = 0.812, *T*_max_ = 0.950	*k* = −16→25
15819 measured reflections	*l* = −19→19

### Refinement

**Table:**

Refinement on *F*^2^	Primary atom site location: structure-invariant direct methods
Least-squares matrix: full	Secondary atom site location: difference Fourier map
*R*[*F*^2^ > 2σ(*F*^2^)] = 0.040	Hydrogen site location: inferred from neighbouring sites
*wR*(*F*^2^) = 0.101	H atoms treated by a mixture of independent and constrained refinement
*S* = 1.04	*w* = 1/[σ^2^(*F*_o_^2^) + (0.0482*P*)^2^ + 0.4941*P*] where *P* = (*F*_o_^2^ + 2*F*_c_^2^)/3
4183 reflections	(Δ/σ)_max_ = 0.001
211 parameters	Δρ_max_ = 0.51 e Å^−^^3^
0 restraints	Δρ_min_ = −0.23 e Å^−^^3^

### Special details

**Table:**

Geometry. All e.s.d.'s (except the e.s.d. in the dihedral angle between two l.s. planes) are estimated using the full covariance matrix. The cell e.s.d.'s are taken into account individually in the estimation of e.s.d.'s in distances, angles and torsion angles; correlations between e.s.d.'s in cell parameters are only used when they are defined by crystal symmetry. An approximate (isotropic) treatment of cell e.s.d.'s is used for estimating e.s.d.'s involving l.s. planes.
Refinement. Refinement of *F*^2^ against ALL reflections. The weighted *R*-factor *wR* and goodness of fit *S* are based on *F*^2^, conventional *R*-factors *R* are based on *F*, with *F* set to zero for negative *F*^2^. The threshold expression of *F*^2^ > σ(*F*^2^) is used only for calculating *R*-factors(gt) *etc*. and is not relevant to the choice of reflections for refinement. *R*-factors based on *F*^2^ are statistically about twice as large as those based on *F*, and *R*- factors based on ALL data will be even larger.

### Fractional atomic coordinates and isotropic or equivalent isotropic displacement parameters (Å^2^)

**Table:**

	*x*	*y*	*z*	*U*_iso_*/*U*_eq_	
S1	0.08445 (7)	0.29509 (2)	0.51315 (3)	0.02175 (10)	
Cl1	0.74643 (8)	0.58625 (3)	1.01336 (3)	0.03431 (13)	
O1	0.71079 (18)	0.45044 (6)	0.51237 (8)	0.0202 (2)	
N1	0.9178 (2)	0.52737 (7)	0.61568 (10)	0.0192 (3)	
N2	0.3993 (2)	0.43359 (7)	0.68077 (9)	0.0169 (2)	
N3	0.3263 (2)	0.39602 (7)	0.60374 (9)	0.0176 (2)	
N4	0.0298 (2)	0.34863 (7)	0.69041 (9)	0.0162 (2)	
C1	0.7024 (2)	0.51529 (8)	0.74926 (11)	0.0175 (3)	
C2	0.6515 (3)	0.52627 (8)	0.84293 (11)	0.0202 (3)	
H2A	0.5189	0.5055	0.8692	0.024*	
C3	0.8066 (3)	0.56950 (9)	0.89592 (11)	0.0213 (3)	
C4	1.0055 (3)	0.60157 (9)	0.85810 (12)	0.0226 (3)	
H4A	1.1051	0.6301	0.8960	0.027*	
C5	1.0559 (3)	0.59112 (9)	0.76381 (12)	0.0218 (3)	
H5A	1.1876	0.6125	0.7376	0.026*	
C6	0.9031 (2)	0.54775 (8)	0.71060 (11)	0.0177 (3)	
C7	0.7407 (2)	0.48082 (8)	0.58967 (11)	0.0176 (3)	
C8	0.5894 (2)	0.47276 (8)	0.67398 (10)	0.0164 (3)	
C9	0.1396 (2)	0.34679 (8)	0.60775 (10)	0.0163 (3)	
C10	−0.1615 (2)	0.30657 (8)	0.72387 (10)	0.0158 (3)	
C11	−0.3209 (2)	0.26888 (8)	0.66549 (11)	0.0175 (3)	
H11A	−0.3036	0.2690	0.6007	0.021*	
C12	−0.5073 (3)	0.23099 (8)	0.70597 (12)	0.0205 (3)	
H12A	−0.6127	0.2050	0.6675	0.025*	
C13	−0.5381 (3)	0.23133 (9)	0.80179 (12)	0.0227 (3)	
H13A	−0.6635	0.2061	0.8277	0.027*	
C14	−0.3798 (3)	0.26976 (10)	0.85915 (12)	0.0248 (3)	
H14A	−0.4001	0.2707	0.9237	0.030*	
C15	−0.1915 (3)	0.30674 (9)	0.82049 (11)	0.0212 (3)	
H15A	−0.0849	0.3318	0.8594	0.025*	
H1N1	1.030 (4)	0.5395 (14)	0.5766 (17)	0.049 (7)*	
H1N3	0.406 (4)	0.3978 (11)	0.5537 (15)	0.029 (5)*	
H1N4	0.095 (3)	0.3761 (11)	0.7308 (14)	0.026 (5)*	

### Atomic displacement parameters (Å^2^)

**Table:**

	*U*^11^	*U*^22^	*U*^33^	*U*^12^	*U*^13^	*U*^23^
S1	0.02155 (19)	0.0258 (2)	0.01799 (19)	−0.00283 (14)	0.00246 (14)	−0.00359 (14)
Cl1	0.0431 (3)	0.0386 (3)	0.0214 (2)	−0.01947 (19)	0.00487 (17)	−0.00397 (16)
O1	0.0156 (5)	0.0242 (5)	0.0208 (5)	0.0009 (4)	0.0028 (4)	0.0007 (4)
N1	0.0136 (6)	0.0218 (6)	0.0225 (7)	−0.0018 (5)	0.0038 (5)	0.0018 (5)
N2	0.0141 (5)	0.0175 (6)	0.0192 (6)	0.0004 (4)	0.0003 (5)	0.0011 (5)
N3	0.0148 (6)	0.0202 (6)	0.0178 (6)	−0.0015 (4)	0.0032 (5)	0.0001 (5)
N4	0.0140 (5)	0.0193 (6)	0.0154 (6)	−0.0035 (4)	0.0003 (4)	−0.0013 (5)
C1	0.0140 (6)	0.0153 (6)	0.0233 (8)	−0.0001 (5)	0.0017 (5)	0.0022 (5)
C2	0.0174 (7)	0.0198 (7)	0.0235 (8)	−0.0035 (5)	0.0021 (6)	0.0023 (6)
C3	0.0231 (7)	0.0199 (7)	0.0209 (7)	−0.0022 (6)	0.0007 (6)	0.0009 (6)
C4	0.0200 (7)	0.0200 (7)	0.0277 (8)	−0.0032 (5)	−0.0023 (6)	0.0022 (6)
C5	0.0144 (6)	0.0222 (7)	0.0288 (8)	−0.0025 (5)	0.0025 (6)	0.0016 (6)
C6	0.0136 (6)	0.0166 (6)	0.0231 (7)	0.0017 (5)	0.0033 (5)	0.0026 (5)
C7	0.0126 (6)	0.0182 (7)	0.0221 (7)	0.0028 (5)	0.0027 (5)	0.0046 (5)
C8	0.0127 (6)	0.0179 (7)	0.0188 (7)	0.0010 (5)	0.0018 (5)	0.0026 (5)
C9	0.0132 (6)	0.0174 (6)	0.0183 (7)	0.0014 (5)	−0.0007 (5)	0.0023 (5)
C10	0.0118 (6)	0.0165 (6)	0.0192 (7)	0.0003 (5)	0.0002 (5)	0.0017 (5)
C11	0.0136 (6)	0.0188 (7)	0.0200 (7)	0.0008 (5)	−0.0017 (5)	−0.0002 (5)
C12	0.0136 (6)	0.0180 (7)	0.0298 (8)	−0.0002 (5)	−0.0025 (6)	0.0007 (6)
C13	0.0143 (6)	0.0245 (8)	0.0295 (9)	−0.0013 (5)	0.0026 (6)	0.0059 (6)
C14	0.0187 (7)	0.0364 (9)	0.0192 (8)	−0.0017 (6)	0.0023 (6)	0.0058 (7)
C15	0.0154 (7)	0.0301 (8)	0.0182 (7)	−0.0038 (6)	−0.0011 (5)	0.0005 (6)

### Geometric parameters (Å, °)

**Table:**

S1—C9	1.6606 (15)	C3—C4	1.392 (2)
Cl1—C3	1.7416 (17)	C4—C5	1.393 (2)
O1—C7	1.2374 (18)	C4—H4A	0.9300
N1—C7	1.3573 (19)	C5—C6	1.383 (2)
N1—C6	1.405 (2)	C5—H5A	0.9300
N1—H1N1	0.88 (3)	C7—C8	1.502 (2)
N2—C8	1.2992 (18)	C10—C15	1.391 (2)
N2—N3	1.3462 (18)	C10—C11	1.395 (2)
N3—C9	1.3872 (18)	C11—C12	1.398 (2)
N3—H1N3	0.85 (2)	C11—H11A	0.9300
N4—C9	1.3467 (19)	C12—C13	1.380 (2)
N4—C10	1.4190 (18)	C12—H12A	0.9300
N4—H1N4	0.84 (2)	C13—C14	1.389 (2)
C1—C2	1.386 (2)	C13—H13A	0.9300
C1—C6	1.409 (2)	C14—C15	1.388 (2)
C1—C8	1.456 (2)	C14—H14A	0.9300
C2—C3	1.389 (2)	C15—H15A	0.9300
C2—H2A	0.9300		
			
C7—N1—C6	111.28 (13)	O1—C7—N1	126.99 (14)
C7—N1—H1N1	121.6 (16)	O1—C7—C8	126.77 (13)
C6—N1—H1N1	127.0 (16)	N1—C7—C8	106.23 (13)
C8—N2—N3	117.04 (13)	N2—C8—C1	125.93 (14)
N2—N3—C9	120.67 (13)	N2—C8—C7	127.47 (14)
N2—N3—H1N3	120.1 (14)	C1—C8—C7	106.56 (12)
C9—N3—H1N3	118.8 (14)	N4—C9—N3	113.18 (13)
C9—N4—C10	131.11 (13)	N4—C9—S1	129.76 (11)
C9—N4—H1N4	113.9 (14)	N3—C9—S1	117.06 (11)
C10—N4—H1N4	114.6 (14)	C15—C10—C11	119.87 (13)
C2—C1—C6	120.49 (14)	C15—C10—N4	116.35 (13)
C2—C1—C8	133.22 (13)	C11—C10—N4	123.73 (13)
C6—C1—C8	106.28 (13)	C10—C11—C12	118.89 (14)
C1—C2—C3	117.25 (14)	C10—C11—H11A	120.6
C1—C2—H2A	121.4	C12—C11—H11A	120.6
C3—C2—H2A	121.4	C13—C12—C11	121.37 (14)
C2—C3—C4	122.48 (15)	C13—C12—H12A	119.3
C2—C3—Cl1	118.78 (12)	C11—C12—H12A	119.3
C4—C3—Cl1	118.71 (12)	C12—C13—C14	119.25 (14)
C3—C4—C5	120.36 (15)	C12—C13—H13A	120.4
C3—C4—H4A	119.8	C14—C13—H13A	120.4
C5—C4—H4A	119.8	C15—C14—C13	120.28 (15)
C6—C5—C4	117.56 (14)	C15—C14—H14A	119.9
C6—C5—H5A	121.2	C13—C14—H14A	119.9
C4—C5—H5A	121.2	C14—C15—C10	120.34 (14)
C5—C6—N1	128.57 (14)	C14—C15—H15A	119.8
C5—C6—C1	121.85 (15)	C10—C15—H15A	119.8
N1—C6—C1	109.57 (13)		
			
C8—N2—N3—C9	173.15 (13)	C6—C1—C8—N2	179.26 (14)
C6—C1—C2—C3	−0.6 (2)	C2—C1—C8—C7	−177.07 (15)
C8—C1—C2—C3	177.83 (15)	C6—C1—C8—C7	1.51 (15)
C1—C2—C3—C4	0.5 (2)	O1—C7—C8—N2	−1.1 (2)
C1—C2—C3—Cl1	178.76 (11)	N1—C7—C8—N2	179.62 (14)
C2—C3—C4—C5	0.1 (2)	O1—C7—C8—C1	176.64 (14)
Cl1—C3—C4—C5	−178.24 (12)	N1—C7—C8—C1	−2.68 (15)
C3—C4—C5—C6	−0.4 (2)	C10—N4—C9—N3	−178.02 (13)
C4—C5—C6—N1	−178.16 (14)	C10—N4—C9—S1	1.7 (2)
C4—C5—C6—C1	0.3 (2)	N2—N3—C9—N4	7.47 (19)
C7—N1—C6—C5	176.63 (15)	N2—N3—C9—S1	−172.31 (10)
C7—N1—C6—C1	−1.99 (16)	C9—N4—C10—C15	162.39 (15)
C2—C1—C6—C5	0.2 (2)	C9—N4—C10—C11	−20.3 (2)
C8—C1—C6—C5	−178.57 (13)	C15—C10—C11—C12	−0.8 (2)
C2—C1—C6—N1	178.95 (13)	N4—C10—C11—C12	−178.04 (13)
C8—C1—C6—N1	0.15 (15)	C10—C11—C12—C13	1.1 (2)
C6—N1—C7—O1	−176.46 (14)	C11—C12—C13—C14	−0.4 (2)
C6—N1—C7—C8	2.86 (16)	C12—C13—C14—C15	−0.6 (2)
N3—N2—C8—C1	−177.80 (13)	C13—C14—C15—C10	0.9 (2)
N3—N2—C8—C7	−0.5 (2)	C11—C10—C15—C14	−0.2 (2)
C2—C1—C8—N2	0.7 (3)	N4—C10—C15—C14	177.23 (14)

### Hydrogen-bond geometry (Å, °)

**Table:**

*D*—H···*A*	*D*—H	H···*A*	*D*···*A*	*D*—H···*A*
N1—H1N1···O1^i^	0.89 (2)	1.98 (2)	2.8560 (17)	171 (2)
N3—H1N3···O1	0.86 (2)	2.08 (2)	2.7563 (16)	135.9 (19)
N4—H1N4···N2	0.84 (2)	2.156 (18)	2.6099 (17)	113.8 (16)
C2—H2A···Cl1^ii^	0.93	2.81	3.6935 (17)	158
C11—H11A···S1	0.93	2.61	3.2423 (14)	126

Symmetry codes: (i) −*x*+2, −*y*+1, −*z*+1; (ii) −*x*+1, −*y*+1, −*z*+2.
